# Developmental Enamel Defects in Children Prenatally Exposed to Anti-Epileptic Drugs

**DOI:** 10.1371/journal.pone.0058213

**Published:** 2013-03-08

**Authors:** Pernille E. Jacobsen, Tine B. Henriksen, Dorte Haubek, John R. Østergaard

**Affiliations:** 1 Section of Pediatric Dentistry, Department of Dentistry, Health, Aarhus University, Aarhus, Denmark; 2 Perinatal Epidemiology Research Unit, Department of Pediatrics, Aarhus Universty Hospital, Aarhus, Denmark; 3 Center for Rare Diseases, Department of Pediatrics, Aarhus University Hospital, Aarhus, Denmark; University of Toronto, Canada

## Abstract

**Objective:**

Some anti-epileptic drugs (AED) have well-known teratogenic effects. The aim of the present study was to elucidate the effect of prenatal exposure to AED and the risk of enamel defects in the primary and permanent dentition.

**Methods:**

A total of 38 exposed and 129 non-exposed children, 6–10 years of age, were recruited from the Aarhus Birth Cohort and the Department of Neurology, Viborg Regional Hospital, Denmark. Medication during pregnancy was confirmed by the Danish Prescription Database. All children had their teeth examined and outcomes in terms of enamel opacities and enamel hypoplasia were recorded.

**Results:**

Children prenatally exposed to AED have an increased prevalence of enamel hypoplasia (11% *vs*. 4%, odds ratio (OR) = 3.6 [95% confidence interval (CI): 0.9 to 15.4]), diffuse opacities (18% *vs*. 7%, OR = 3.0; [95% CI: 1.0 to 8.7, p<0.05]), and numerous (>3) white opacities (18% *vs*. 10%, OR = 2.2; [95% CI: 0.8 to 6.1]) in the primary dentition. In the permanent dentition, an increased risk of numerous (>3) white opacities (34% *vs.* 12%, OR = 3.3; [95% CI: 1.3 to 8.4]) was found.

**Conclusions:**

The present study shows that children prenatally exposed to AED have an increased risk of developing numerous teeth with white opacities in their primary and permanent dentition. In addition, they also have an increased risk of developing diffuse opacities and enamel hypoplasia in their primary teeth.

## Introduction

**Figure 1:**
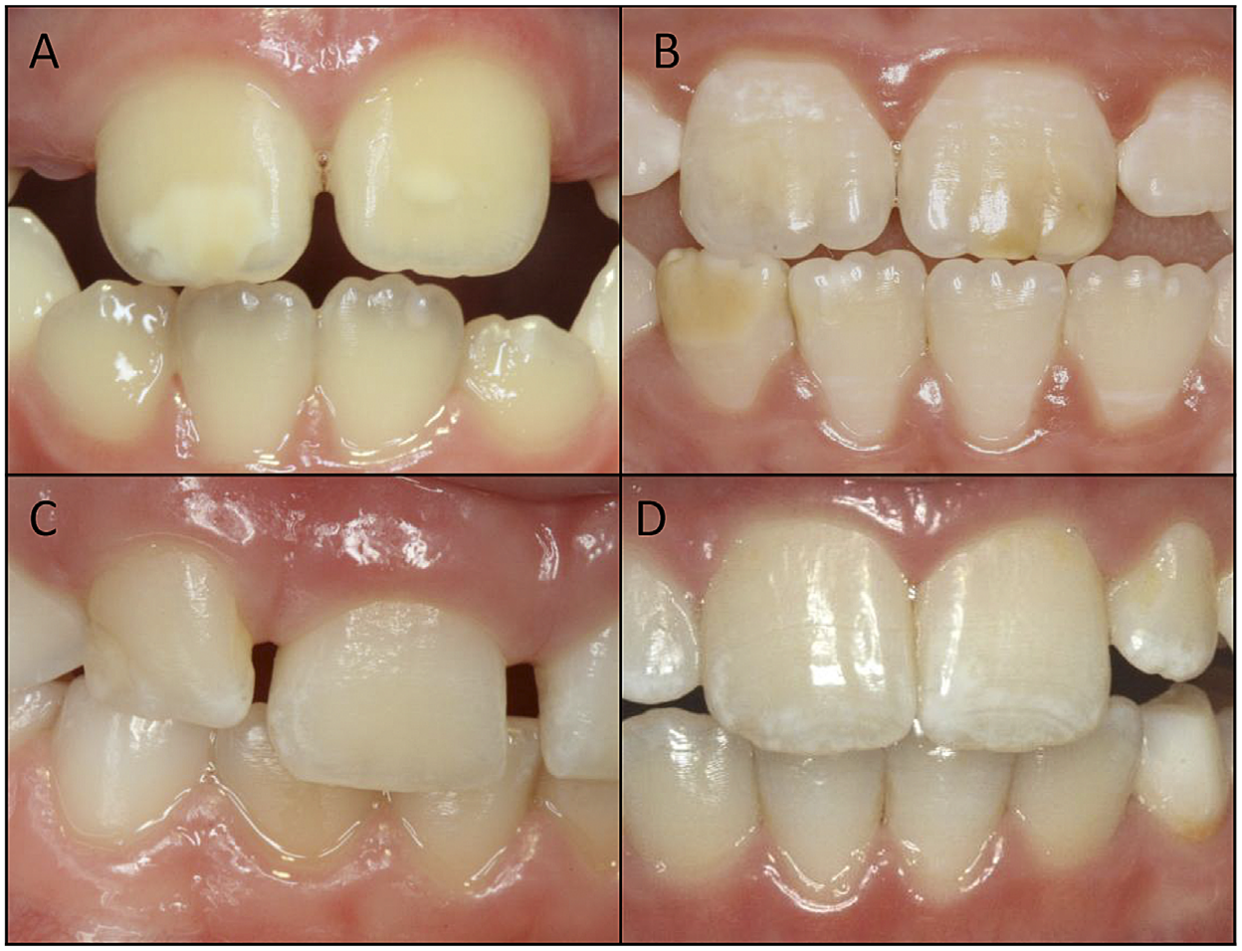
Different Enamel Defects on the Upper Permanent Incisors. A) white/yellow demarcated opacities B) yellow/brown opacities C) enamel hypoplasia D) diffuse opacities.

Disturbances in the development of enamel may involve genetic, systemic, local or environmental factors, among others. The severity and extent of the enamel defects varies, depending on which factors are involved. Defects range from severe enamel loss of all teeth present to local, demarcated opacities on a single tooth. The enamel morphogenesis initiates with secretion of enamel matrix proteins followed by mineralization and finally maturation. The enamel morphogenesis is a continuous, complex process starting with the development of the cusps on the molars and the incisal part of the incisors, followed by the complete enamel formation reaching the cervical area of the tooth. The enamel of the primary teeth begins to develop week 12 postconceptually and gradually continues its development throughout the whole pregnancy. The time interval for enamel development on the permanent teeth starts in week 28 and begins to mineralize at the time of birth. The permanent incisors and first permanent molar complete their mineralization during the first three years of life [1]. Disturbances in different stages of the enamel formation may result in different outcomes, depending on the time frame of the exposure, and the impact mechanisms of the factor(s) involved. Enamel malformation can be subdivided into demarcated opacities, diffuse opacities or hypoplasia (Fig. 1).

Previous investigations have documented that systemic influence of chemical substances like fluoride, dioxin, or tetracycline has teratogenic effects on the fetus [2]–[6], and exposure during the enamel morphogenesis will result in inadequate enamel formation [6]. Anti-epileptic drugs (AED) are also known to be teratogenic; and depending on the type of drug, number of drugs, and dose, children exposed to AED prenatally have a two to three times higher risk of developing congenital abnormalities [7], [8]. So far, the impact of AED on enamel morphogenesis has not been studied, but the development of enamel is, indeed, at risk of teratogenic influence of AED.

The aims of the present study are to elucidate the occurrence and the risk of developing opacities and hypoplasia in primary and permanent teeth in children exposed to AED prenatally.

## Materials and Methods

Both children exposed to AED prenatally and unexposed children were recruited from the Aarhus Birth Cohort. Children exposed to AED were furthermore recruited from the Department of Neurology, Viborg Regional Hospital, Denmark. In short, information on alcohol intake, smoking, and medication during pregnancy are registered in The Aarhus Birth Cohort. Structured information about the newborn child, such as gestational age, birth weight and Apgar score are also recorded. For a detailed description of the cohort, see Hedegaard et al. [9]. The Department of Neurology, Viborg Regional Hospital participates in the European and International Registry of Antiepileptic Drugs in Pregnancy [10] and therefore has specific data on pregnant women receiving antiepileptic medicine. The mother’s medical chart contains information on medical use during pregnancy and pregnancy outcome, but no information on other lifestyle habits of the mother is available. During pregnancy, seizure control was monitored closely and AED dose were regulated in order to prevent seizures.

Children between 6–10 years of age were enrolled in the study. Twins and children with a birth weight below 2500 g were excluded. In addition, children prenatally exposed to other potentially teratogenic drugs, such as anti-depressive or anti-psychotic drugs, were also excluded. A total of 87 exposed children were available in the two registers: 53 were recruited from the Aarhus Birth Cohort and 34 were recruited from The Department of Neurology, Viborg.

Based on an estimate of a three times higher prevalence rate of 17% in exposed than in unexposed children, and statistical significance defined as a two-sided p-value below 5%, and a power of 100%, we estimated that the number of controls should be approximately three times the number of the exposed children. Children within the same age range, with similar gestational age and birth weight range, but unexposed to AED, were selected randomly from the Aarhus Birth Cohort as control group (unexposed children). The intake of medication during pregnancy and post partum was verified by data from the Danish Prescription Database [11].

A total of 87 exposed and 298 unexposed children were invited to participate in the study. Invitations with information on the study and a pre-paid return envelope were posted to the parents. Two reminders were sent out in case of no response. If parents agreed to participate, a written consent was signed and the child was examined. The study was approved by the Central Denmark Region Committees on Health Research Ethics (j.nr. 20100028).

All children were examined by the first author (PEJ). PEJ had been trained by an experienced dentist who had previously performed similar registration [12]. The training consisted of clinical photos showing the different outcomes and clinical examination of voluntary children with the different outcomes present. The dental examinations were performed by use of light, mirrors, and cotton rolls to dry the teeth. Subsequently, a clinical photograph was taken. PEJ was blinded concerning the child’s exposure. The outcome measurements were defined based on the modified DDE index (Developmental Defects of Enamel) [13] and the conditions were defined and recorded as follows.

- Status: the status of each tooth was recorded as permanent, primary, or missing
- Eruption stage: if the crown was more than half-erupted, the tooth was recorded as fully erupted; otherwise, partly erupted or not visible
- Demarcated opacities: are localized qualitative defects obtained by an incomplete mineralization, resulting in lack of normal coloring and translucency of the enamel. The opacities were recorded either as none, white/yellow or yellow/brown lesions. In case of doubt, the less severe condition was recorded.
- Diffuse opacities: are scattered qualitative defects obtained by an incomplete mineralization, resulting in lack of normal coloring and translucency of the enamel. The diffuse opacities were recorded either as none or present.
- Hypoplasia: is defined as quantitative loss of enamel due to deficient enamel matrix formation and could be recorded with or without concomitant presence of opacities.

A tooth was recorded with one score per outcome, only. Thus, outcome measurement reflects the number of teeth affected and not number of enamel defects.

### Intra-examiner Validation

To evaluate the reproducibility of the method used, 10% of the children were randomly chosen for re-examination. Kappa values were calculated including all teeth fully erupted at both examination dates.

In the permanent dentition, the kappa values were 0.7 and 0.8 for the white and yellow demarcated opacities, and 0.6 and 0.9 for diffuse opacities and hypoplasia, respectively. In the primary dentition, the kappa values were 0.6 for the demarcated as well as for diffuse opacities. No hypoplasia was present in the primary teeth examined.

### Statistics

All data were entered in EpiData and exported to STATA for analyses. Student’s t-test was used to test differences between continuous variables and for categorized variables; Chi-square test for independence within contingency tables was used. A two sided p-value below 0.05 was considered statistically significant. Analyses of the association between the exposure and the binary outcome variables were performed by use of simple logistic regression. The odds ratios (OR) and 95% confidence intervals (CI) were adjusted for clustering among individuals by the *vce* syntax in STATA. In addition, multiple logistic regression analysis was performed to analyze if gender, smoking or use of antibiotics should be considered confounders. Subsequently, sub-analysis on the therapeutic effect was performed by the same method as described above.

## Results

A total of 38 exposed and 129 non-exposed children were included in the study. The participation rate was 48% and similar for both groups. Table 1 shows the characteristics of the participants and non-participants. No significant differences between the participants and the non-participants in any of the listed parameters were found. In addition, there were no significant differences between the exposed and unexposed participants in the study. The children were exposed to different kind of AED such as lamotrigine, carbamazepine, and valproate among others. A total of 6 children were exposed to polytherapy and half the children were exposed to lamotrigine only. We have no information about seizure control during pregnancy or measurements of blood concentrations of AEDs. None of the children enrolled in the study suffered from any syndromes or genetic diseases that may be related to dental abnormalities. Table 2 summarizes the distribution of the different enamel defects. The number of children with more than two teeth with white opacities was significantly higher in the children exposed to AED *in utero* than among the unexposed children. This was found for primary teeth (18% *vs.* 10%) as well as for permanent teeth (34% *vs.* 12%). In addition, children with fetal exposure to AED had an increased prevalence of diffuse opacities (18% *vs.* 7%) and enamel hypoplasia (11% *vs.* 3%) in their primary dentition. However, in the permanent teeth of unexposed children we found an increased prevalence of children with yellow opacities (11% *vs.*18%).

**Table 1:** Characteristics of women-offspring dyads by whether they were included or dropouts.

	Included		Drop outs	
	Exposed	Not exposed	Exposed	Not exposed
Number of children	38	129	44	156
Antimicrobials^a^	17	72	21	77
Analgesics^a^	4	22	13	30
Antihistamins^a^	5	4	4	9
Others topical	20	63	26	78
Others systemic^b^	8	4	7	2
Gestational age mean^c^ (SD)	39+5 (1+3)	39+5 (1+4)	39+1 (1+6)	39+5 (1+3)
Birth weight, mean (SD)	3636 (500)	3664 (491)	3585 (489)	3470 (523)
Smoking, (n/total n) (%)^d^	(3/20) 15	(10/127) 8	(5/27) 19	(17/155) 11

a Number of women receiving a prescription and relate to a period 1 year before birth to 1 year after.

b Others refer to prescriptions on drugs for topical use, such as Spirocort® or Brentan® and other medication for systemic use, such as propranolol or Klexane®.

c Gestational mean age is given as weeks+days.

d Data on smoking habits is obtained from the Aarhus Birth Cohort only.

**Table 2:** Number of children with the number of teeth affected by the different enamel defects by whether they were exposed to antiepileptic drugs during fetal life and whether the findings were in primary or permanent teeth.

		Primary teeth		Permanent teeth^a^	
		Exposed	Not exposed	Exposed	Not exposed
Number of children		38	128	38	124
White opacities, n(%)	0	20 (53)	80 (63)	17 (45)	66 (53)
	1–2	11 (29)	35 (27)	8 (21)	43 (35)
	3+	7 (18)	13 (10)	13 (34)	15 (12)
Yellow opacities, n(%)	0	33(87)	115 (90)	34 (89)	102 (82)
	1+	5 (13)	13 (10)	4 (11)	22 (18)
Diffuse opacities, n(%)	0	31 (82)	119 (93)	22 (58)	74 (60)
	1+	7 (18)	9 (7)	16 (42)	50 (40)
Hypoplasia, n(%)	0	34 (89)	124 (97)	35 (92)	117 (94)
	1+	4 (11)	4 (3)	3 (8)	7 (6)

a The permanent teeth only involve the incisors and 1^st^ molar.

Table 3 shows odds ratios for enamel defects in the primary or permanent teeth for exposed and unexposed children. Children exposed to AED *in utero* had an increased risk of developing more than two white opacities in the primary (OR = 2.2; CI 0.8–6.1) as well in the permanent teeth (OR = 3.3; CI 1.3–8.4) compared with unexposed children. However, when exposed to lamotrigine only, we found no increase in risk for white opacities in primary or permanent dentition. The risk for white opacities was limited to the offspring of the mothers who had received more than one AED or AEDs different from lamotrigine.

**Table 3:** The odds ratios and confidence intervals of the number of teeth affected by the different enamel defects and by whether the children were exposed or not, during fetal life and whether the findings were in primary or permanent teeth.

		Primary teeth				Permanent teeth^a^			
		Exposed	Only lamotrigine^b^	Other^c^	Exposed		Only lamotrigine^b^	Other^c^	
Number of children		38	19	19	38		19	19	
White opacities, OR (95% CI)	1–2	1.3 (0.5–2.9)	1.2 (0.4–3.7)	1.2 (0.4–4.1)	0.7 (0.3–1.8)		0.2 (0.1–1.1)	2.3 (0.6–8.7)	
	3+	2.2 (0.8–6.1)	1.1 (0.2–5.7)	3.4 (1.0–11.9)^	3.3 (1.3–8.4)**		1.4 (0.4–4.8)	9.9 (2.7–36.7)***	
Yellow opacities, OR (95% CI)	1+	1.3 (0.4–4.0)	1.0 (0.2–5.0)	1.7 (0.4–6.5)	0.5 (0.2–1.7)		0.9 (0.2–3.3))	0.3 (0.0–2.0)	
Diffuse opacities, OR (95% CI)	1+	3.0 (1.0–8.7)*	2.5 (0.6–10.2)	3.5 (1.0–13.0)^	1.1 (0.5–2.3)		1.1 (0.4–2.9)	1.1 (0.4–2.9)	
Hypoplasia, OR (95% CI)	1+	3.6 (0.9–15.4)	5.8 (1.2–28.5)*	1.7 (0.2–16.4)	1. 1.4 (0.4–5.9)		2.0 (0.4–10.3)	0.9 (0.1–8.1)	

a The permanent teeth only involve the incisors and 1^st^ molars.

b Include children who only have been exposed to lamotrigine for the whole period.

c Include children, who have been exposed to poly pharmacy, other anti-epileptic drugs than lamotrigine or a mix of anti-epileptic drugs, including lamotrigine.

^ Not significant despite confidence interval larger than 1.

* p<0.05;

** p<0.01;

*** p<0.001.

We found no differences in the occurrence of yellow opacities within the two groups.

As regards diffuse opacities, we only found an increased risk within the primary teeth of children exposed to AED (OR = 3.0; CI 1.0–8.7; p<0.05), and this risk was restricted to offspring exposed to AED other than lamotrigine or more than one kind of AED.

Concerning enamel hypoplasias, we also found that the increased risk was restricted to the primary teeth of children exposed to AED (OR = 3.6; CI 0.9–15.4). However, the offspring exposed to lamotrigine had a statistically significant increase only of enamel hypoplasias compared with the unexposed children (OR = 5.8; CI 1.2–28.5).

## Discussion

We have shown that children with prenatal exposure to anti-epileptic drugs have an increased risk of developmental enamel defects, *i.e.* opacities and hypoplasia. A more specific analysis showed that children exposed to lamotrigine only were at increased risk of hypoplasia in their primary teeth, whereas children exposed to other anti-epileptic drugs than lamotrigine or more than one AED had an increased risk of demarcated opacities.

To our knowledge, this is the first study of anti-epileptic drug exposure during fetal life and tooth development. Regardless of the low participant rate, the study has a high power (98%). The description of the withdrawals (Table 1) shows that participants and non-participants are quite similar, which indicates that the children enrolled in the study may be representative. On the other hand, we cannot exclude the risk of selection bias, because the participants had to come to the dental school for the examination and the most common reasons not to participate in the study were lack of transportation, lack of time, or too long transportation time. We were able to eliminate some of the potential confounders prior to the inclusion, but we had no access to comprehensive information about social status, education level, or coffee intake, which might be of importance to the risk of confounding. Analyses showed similar results for boys and girls, but due to insufficient information on smoking habits, we were not able to conduct valid analyses on this possible confounder. The number of participants excluded further sub-analysis, such as analyses of more specific AED exposures and dose response relationships. Such information could, however, have been informative, especially because the present study suggests that there is a difference in relation to the different drugs metabolic influence on the enamel development.

White opacities are the most frequent outcome recorded in the present study. Nearly half of the children enrolled in the study had one or more teeth with white opacities. The general consideration concerning the etiology of opacities is that the tooth morphogenesis is affected due to a harmful exposure(s) during its mineralization phase. The fact that the children exposed to antiepileptic drugs have an increased risk of developing several opacities in their dentition implies that the exposure is scattered over a larger period of time and may therefore influence the development of several teeth. We found this to apply to both the primary and the permanent teeth, indicating that the concentration may remain high enough during breastfeeding to influence the mineralization of the enamel in the permanent teeth, which mainly occurs after birth. Why this only applies to the children who at one point have been exposed to other anti-convulsants than lamotrigine is unclear, but it has been shown that anti-epileptic drugs, such as valproic acid and oxcarbazepine, induce lower bone density and involve an increased risk of fracture [14] in patients with epilepsy, and that patients treated with AED have lower blood calcium levels than patients who are not treated with AED [15]. To our knowledge, no studies have found abnormalities in bone metabolism in patients receiving lamotrigine as mono therapy. Along with other minerals, calcium is crucial for development of normally mineralized enamel, so opacities in offspring exposed to other AED than lamotrigine could be due either to the low calcium level in the mother or to a more directly teratogenic effect of the AED exposure.

Yellow opacities are more severe defects due to extensive hypomineralization of the enamel. This condition can be cosmetically inappropriate and even painful because the enamel has a tendency to break off. We found no association between prenatal exposure to AED drugs and yellow opacities. Conversely, it seems that the exposed children have a decreased risk. Yellow opacities are very visible and might even be accompanied by pain in the children involved; the number of children with yellow opacities may therefore be over-represented, especially in unexposed children because the reason(s) for entering the study presumably are different in the two groups. This suspicion of self-selection is supported, when comparing with other studies indicating a much lower prevalence of yellow opacities than the prevalence found in the present study [12], [16].

Diffuse opacities are a result of continuous, harmful exposure over a longer period of time. We found a higher prevalence of diffuse opacities in the primary teeth in exposed compared to non-exposed children, but not in the permanent teeth. When exposed to AED through breast-feeding, the concentration of anti-epileptic medicine is not as high as during pregnancy; and even with a risk of accumulation due to the decreased elimination in the newborn infant [17], the exposure may not be sufficiently high to disturb the mineralization of the permanent teeth in a more continuous manner. The overall prevalence of diffuse opacities in the permanent teeth was quite high, which indicates that other factors than just exposure to AED may be of importance. The defects were often located to the incisal part of the tooth crown, which suggests that the teeth are in some way influenced by the mother’s lifestyle habits and affected through the nursing period, since these teeth mineralize during this particular period. However, in some cases, the defects were scattered all over the vestibular surface of the tooth, which indicates that the child may have been continuously exposed to some kind of harmful substance for several years. The only well-known risk factor for diffuse opacities is fluoride consumption [18], but otherwise the etiology remains very unclear. AED does not seem to explain the presence of diffuse opacities in the permanent dentition.

Hypoplasia is a congenital defect of the tooth and develops prior to the phase of hypomineralization. In several studies the defect has been associated with prematurity [19], [20]. In the present study, we eliminated that explanation by excluding all children born with a birth weight below 2500 g. Children exposed to AED had an increased risk of hypoplasia, especially in their primary dentition. In addition, the child at individual level also had more teeth affected than the non-exposed children. More drug-specific analyses showed that lamotrigine may be responsible for this association. In the early stage of enamel formation, the secretory stage, the content of lipid is high. It has been suggested that [21] enamel lipids, especially phospholipid, play a role in tooth development and that alterations could induce enamel hypoplasia. Lamotrigine is lipid-bounded as opposed to many other anti-epileptic drugs that are protein-bound. It can, thus, be speculated that lamotrigine may alter the content or function of enamel lipids in the secretory stage and therefore induces hypoplasia.

In the present study, a considerable number of both exposed and unexposed women were treated with antibiotics. It has previously been discussed whether fever during pregnancy and use of antibiotics might induce birth defects [22], [23]. If this is the case, and use of antibiotics are also associated with antiepileptic treatment, confounding of the association between antiepileptic exposure and enamel defects is possible. However, intake of antibiotics was equally frequent in our two exposure groups and when we adjusted for the use of antibiotics no change in our estimated risks was found. Many other factors have been suspected as risk factors for local enamel defects, mainly in the permanent teeth, including early childhood illness, prolonged nursing period, otitis media, or asthma [24], [25], and we cannot rule out that these factors may be responsible for some of the defects found. However, only if these factors are more common among children of women taking AED this would explain our results. On the contrary, the only well-described factors, responsible for enamel defects in the primary teeth are genetic diseases and prematurity and these factors were excluded prior to the present study. In summary, we conclude that.

- Children prenatally exposed to anti-epileptic drugs other than lamotrigine or lamotrigine in combination with other anti-epileptic drugs have an increased risk of developing multiple white opacities in both their primary and permanent teeth.
- Children prenatally exposed to anti-epileptic drugs other than lamotrigine or lamotrigine in combination with other anti-epileptic drugs have an increased risk of developing diffuse opacities in their primary teeth.
- Children exposed to lamotrigine have an increased risk of developing dental hypoplasia in their primary teeth.
